# Genetic association analysis based on a joint model of gene expression and blood pressure

**DOI:** 10.1186/s12919-016-0045-6

**Published:** 2016-10-18

**Authors:** Stefan Konigorski, Yildiz E. Yilmaz, Tobias Pischon

**Affiliations:** 1 Molecular Epidemiology Research Group, Max Delbrück Center (MDC) for Molecular Medicine, Robert-Rössle-Straße 10, 13125 Berlin, Germany; 2 Department of Mathematics and Statistics, Memorial University of Newfoundland, St. John’s, NL A1C 5S7 Canada; 3 Discipline of Genetics, Faculty of Medicine, Memorial University of Newfoundland, St. John’s, NL A1C 5S7 Canada

## Abstract

Recent work on genetic association studies suggests that much of the heritable variation in complex traits is unexplained, which indicates a need for using more biologically meaningful modeling approaches and appropriate statistical methods. In this study, we propose a biological framework and a corresponding statistical model incorporating multilevel biological measures, and illustrate it in the analysis of the real data provided by the Genetic Analysis Workshop (GAW) 19, which contains whole genome sequence (WGS), gene expression (GE), and blood pressure (BP) data. We investigate the direct effect of single-nucleotide variants (SNVs) on BP and GE, while considering the non-directional dependence between BP and GE, by using copula functions to jointly model BP and GE conditional on SNVs. We implement the method for analysis on a genome-wide scale, and illustrate it within an association analysis of 68,727 SNVs on chromosome 19 that lie in or around genes with available GE measures. Although there is no indication for inflated type I errors under the proposed method, our results show that the association tests have smaller *p* values than tests under univariate models for common and rare variants using single-variant tests and gene-based multimarker tests. Hence, considering multilevel biological measures and modeling the dependence structure between these measures by using a plausible graphical approach may lead to more informative findings than standard univariate tests of common variants and well-recognized gene-based rare variant tests.

## Background

### Biological–statistical framework

**Fig. 1:**
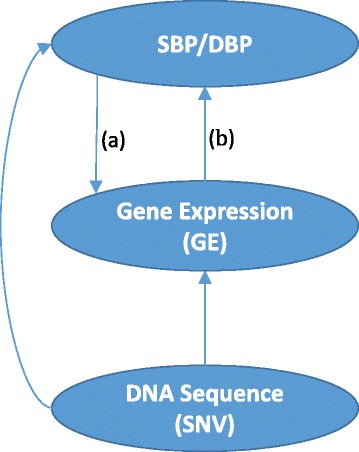
Model of potential relation between DNA sequence, gene expression levels, and blood pressure. DBP, diastolic blood pressure; SBP, systolic blood pressure

In recent years, the further development and refinement of high-throughput technologies has allowed genetic associations to be investigated in more depth. Using whole genome sequence (WGS) data, rare variants are being analyzed with the hope of explaining more of the heritable variation in phenotypes unexplained by common single-nucleotide variants (SNVs). However, the results of recent studies seem to suggest that the success herein has been limited despite the development and use of new gene-based rare-variant tests. We argue that statistical models and methods with a more meaningful and appropriate biological basis are needed to obtain more powerful association tests. Accordingly, we propose a modeling framework incorporating multilevel biological measures in the context of the available Genetic Analysis Workshop (GAW) 19 data and a corresponding statistical method for genetic association analysis of common and rare variants. The arguments in this study build on the biological framework depicted in Fig. 1, which describes the potential effects between the different measured variables.

Empirical evidence exists for effects (a) and (b) in Fig. 1 in inverse directions [1], which suggests non-directional modeling of the association between blood pressure (BP) and gene expression (GE). Accordingly, this study proposes a joint model of BP and GE conditional on SNVs based on copula functions [2] to identify SNVs associated with BP and/or GE while considering non-directional associations between them, and implements the approach so that it can be applied on a genome-wide scale. Previous studies [3] showed that association tests of common variants in copula models have higher power when there is a high correlation between the 2 phenotypes. In this study, we investigate whether or not this improvement holds true under moderate or low correlations as well, and whether or not there is any power gain in single rare-variant association tests when incorporating multilevel biological measures. Copula functions are used to construct a joint distribution by combining the marginal distributions with a dependence structure. In the copula modeling approach, the parameters of the dependence between BP and GE do not appear in the marginal distributions. This property is very useful in estimating the direct effects of SNVs on BP or GE, while considering the dependence between BP and GE, and also in identifying pleiotropic SNVs which are associated with both phenotypes. In addition to identifying SNVs associated with BP and/or GE, the framework also allows, for example, a focus on searching for SNVs explaining the association between GE and BP [3].

## Methods

### Data description

The family data set of the GAW 19 contains information on 157 unrelated individuals from the San Antonio family studies pedigrees, including systolic BP (SBP) and diastolic BP (DBP) measurements, information regarding current use of antihypertensive medication, and non-genetic covariates (sex, age, and current tobacco smoking status) at one or more examination time points [4]. GE measurements in lymphocytes at the first time point are available for 20,634 transcripts, which were already quality-checked, filtered for detectable expression, and quantile-normalized. For 113 unrelated individuals, complete data is available for BP, GE, and the non-genetic covariates, and among them, 81 individuals have WGS data. We conduct genetic association analyses in this subset of 81 unrelated individuals using real phenotypes. The sequence data is available for odd-numbered chromosomes and was first processed before the analysis, with standard quality control checks and the exclusion of SNVs with more than 20 % missing base calls. We focus on the BP measurements and non-genetic covariates at the first time point. Of the 2 BP phenotypes, only SBP is considered.

### Statistical methods

To illustrate and evaluate the copula method, chromosome 19 was analyzed, as it contains the transcript with the highest association with SBP (IL12RB1, Kendall’s *τ* = 0.24, *p* = 2.5 × 10^− 4^) among all 11,542 available mapped transcripts, and we hypothesize that it could lead to the largest power increase under the joint model compared to univariate models. GE was measured for 848 transcripts on chromosome 19. All *N* = 68, 727 non-monomorphic, biallelic SNVs with minor allele frequency (MAF) equal to or greater than 0.015 (i.e., with at least 3 copies of the minor allele) which lie in or within 5 kb of these genes were analyzed in separate copula models of SBP and the corresponding GE. We used the annotations in the provided variant call format (VCF) files, the provided mapping of GE probes to gene names, and the *Ensembl* database with reference genome GRCh37.p13 through *BioMart* to obtain physical positions of genes and SNVs.

In a preparatory step of phenotype definition, we adjusted the SBP and GE measures for the effect of the non-genetic covariates, including antihypertensive medication. Adjusting SBP for the effect of BP-lowering medication is important because the objective is to explain the variation in SBP. Following the method described in Konigorski et al. [3], we fitted a censored regression model conditional on the non-genetic covariates age, sex, and smoking status, with medication use as a censoring indicator. For treated individuals, we estimated the “true” underlying SBP from the conditional expectation of SBP, given that the observed SBP is lower than the true SBP, and also conditional on the non-genetic covariates. For further analysis, adjusted SBP phenotypes *SBP*_*adj*_ are the residuals obtained as follows: an untreated individual’s adjusted SBP is the difference between observed and fitted SBP; a treated individual’s adjusted SBP is the difference between estimated true and fitted SBP. Adjusted GE phenotypes *GE*_*j*,*adj*_, *j* = 1, 2, …, 848, for the following analysis steps are residuals obtained from fitting a linear regression for each *GE*_*j*_ on the nongenetic covariates age, sex, smoking status, and antihypertensive medication.

In the genetic association analysis, we fitted a copula model for the joint distribution of *SBP*_*adj*_ and *GE*_*j*,*adj*_, *j* = 1, 2, …, 848, conditional on each single variant *SEQ*_*i*_ in and around the gene,

1 $$ F\left(SB{P}_{adj},G{E}_{j,adj}\Big|SE{Q}_i\right) = {C}_{\psi}\left({F}_1\left(SB{P}_{adj}\Big|SE{Q}_i\right),{F}_2\left(G{E}_{j,adj}\Big|SE{Q}_i\right)\right) $$

where *C*_*ψ*_ is a copula function with dependence parameter *ψ*, and *F*_1_ and *F*_2_ are the marginal distributions of *SBP*_*adj*_ and *GE*_*j*,*adj*_, respectively, with models

2 $$ SB{P}_{adj} = {\alpha}_0 + {\alpha}_1SE{Q}_i + {\varepsilon}_i $$

3 $$ G{E}_{j,adj} = {\beta}_0 + {\beta}_1SE{Q}_i + {\varepsilon}_i^{\hbox{'}}. $$

We consider the 2-parameter copula family

4 $$ {C}_{\psi}\left({u}_1,{u}_2\right) = {\left\{{\left[{\left({u_1}^{-\varphi }-1\right)}^{\theta } + {\left({u_2}^{-\varphi }-1\right)}^{\theta}\right]}^{1/\theta }+1\right\}}^{-1/\varphi } $$

with 0 ≤ *u*_1_, *u*_2_ ≤ 1, and the copula parameters *ψ* = (*φ*, *θ*), *φ* > 0, *θ* ≥ 1, which allows a flexible modeling of both the lower- and upper-tail dependence and contains a large class of copulas, including the Clayton and the Gumbel-Hougaard copula [2]. To identify SNVs associated with *SBP*_*adj*_ and *GE*_*j*,*adj*_, we tested the null hypotheses *H*_0_ : *α*_1_ = 0 (vs. *H*_*A*_ : *α*_1_ ≠ 0) and *H*_0_ : *β*_1_ = 0 (vs. *H*_*A*_ : *β*_1_ ≠ 0), respectively, by using the large sample Wald test statistics.

For a comparison with univariate approaches, standard linear regression models of *SBP*_*adj*_ in equation (2) and *GE*_*j*,*adj*_ in equation (3) were fitted independently. Aside from the single-variant tests, gene-based association tests for rare variants were also considered. In particular, a variance-component test (the sequence kernel association test; SKAT [5]) and a test that combines a burden and variance-component test (the optimal sequence kernel association test; SKAT-O [6]) were used for comparison. To have the best possible performance of the SKAT and SKAT-O as a reference, all possible kernels, *p* value computation methods, and inclusions of variants (in gene only, in and around gene, rare variants, or rare and common variants) of the *SKAT* function in R were used, and the minimum *p* value of all these tests was extracted for a given gene. To assess the significance of the results corrected for multiple testing, adjusted *p* values were calculated using the R function *p.adjust* with the Benjamini and Hochberg adjustment (BH-adjustment) [7], which controls the false discovery rate.

## Results

Of the *N* = 68, 727 analyzed variants on chromosome 19, 18,916 are rare variants with MAF between 0.015 and 0.05 (inclusive), and the remaining 49,811 variants have a MAF greater than 0.05. On average, 96 SNVs were tested with each GE (some SNVs were tested with more than 1 GE because of gene overlap/proximity). The smallest gene contains 4 SNVs and the largest gene contains 920 SNVs. For model selection, the Akaike Information Criterion (AIC) was computed for each model. We observed that the copula model had a smaller AIC than a bivariate normal model conditional on any SNV; therefore, it has a better model fit. In addition, the copula model had a smaller AIC than the working independence model when there was a moderate association between *SBP*_*adj*_ and *GE*_*j*,*adj*_ (i.e., when the estimated Kendall’s *τ* is greater than 0.11).

**Fig. 2:**
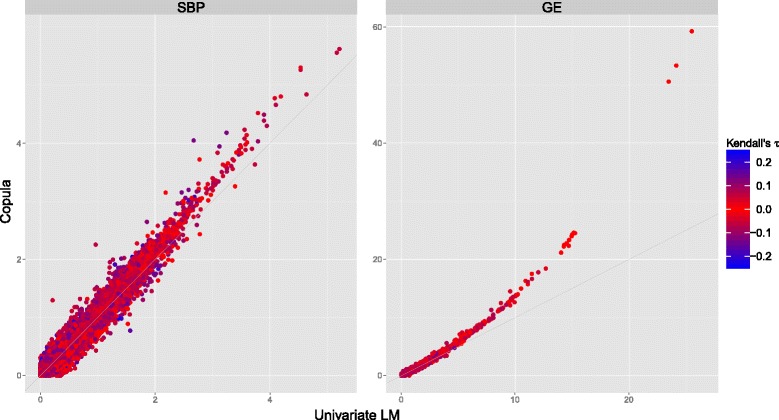
Scatterplots of the *p* values (on a − log_10_ scale) of all tested common and rare SNVs for testing *H*_0_ : *α*_1_ = 0 (for SBP, *left panel*) and *H*_0_ : *β*_1_ = 0 (for GE, *right panel*) under the copula model versus the univariate model. For each SNV, the strength of the association (Kendall’s *τ*) between the corresponding GE and SBP is shown by the *color of the dots*

Based on an *α* level of 0.05 and the adjusted *p* values, 5 SNVs in the gene *CEACAM5* are identified to be significantly associated with SBP under the copula model (Table 1). Additionally, 1075 SNVs in 122 different genes are identified to be significantly associated with their corresponding GE as local *(cis)* expression quantitative trait loci (eQTLs; Table 2). For these significant SNVs, Kendall’s *τ* between the corresponding GE and SBP is estimated as 0.21 or less. In comparison, none of the 68,727 SNVs are identified as significant based on the univariate regression model of SBP (Table 1), and based on the univariate regression model of GE, 800 SNVs in 80 genes are significantly associated with GE (Table 2). The power gain under the copula model compared to the univariate model is shown in Fig. 2, which shows plots of the *p* values of all tested SNVs under the copula model versus the univariate models of SBP and GE. In particular, lower *p* values are much lower under the copula model compared to the univariate models. The power gain can also be obtained when the dependence between SBP and GE is very low, and it is a result of: (a) lower SE estimates of the SNV effect under the copula model, while point estimates are similar and the mean difference between point estimates is 0, and (b) the different (asymptotic) null distribution of the association test statistics (normal vs. t distribution). The top hits under the 2 models are in the same order with respect to *p* values, and consist of rare variants significantly associated with SBP (Table 1), and common variants significantly associated with GE (Table 2).

**Table 1:** Results of the SNVs significantly associated with SBP under the copula model and results under the univariate model

SNV	MAF	Location	Copula			Univariate		
			$\hat{\alpha_1}$ (SE)	*p v*alue	Adj. *p* value	$\hat{\alpha_1}$ (SE)	*p v*alue	Adj. *p* value
rs10402825	0.04	*CEACAM5* (protein coding)	30.77 (6.52)	2.40 × 10^− 6^	4.42 × 10^− 2^	32.21 (6.63)	6.18 × 10^− 6^	1.12 × 10^− 1^
rs7258524	0.04	*CEACAM5* (upstream)	30.75 (6.56)	2.77 × 10^− 6^	4.42 × 10^− 2^	32.28 (6.67)	6.86 × 10^− 6^	1.71 × 10^− 1^

*Adj. p value* is the adjusted *p* value with BH-correction; *MAF* denotes the minor allele frequency in this sample. The estimated Kendall’s *τ* between *SBP*_*adj*_ and *GE*_*CEACAM*5,*adj*_ is − 0.13. Note: The markers are in very high linkage disequilibrium (LD) (r = 0.99). SNVs rs34155934, rs35091611, and rs10415940 in the same gene are in perfect LD with rs7258524 and have identical association

**Table 2:** Results of the SNVs with the smallest *p* values in the association tests of *H*_0_ : *β*_1_ = 0 for GE under the copula and univariate models

SNV	MAF	Location	Copula			Univariate		
			$\hat{\beta_1}$ (SE)	*p v*alue	Adj. *p* value	$\hat{\beta_1}$ (SE)	*p v*alue	Adj. *p* value
rs2314667	0.32	UBA52 (upstream)	0.88 (5.42 × 10^− 02^)	6.47 × 10^− 60^	1.71 × 10^− 55^	0.88 (5.49 × 10^− 02^)	3.21 × 10^− 26^	8.71 × 10^− 22^
rs2314664	0.33	UBA52 (downstream)	0.85 (5.52 × 10^− 02^)	5.01 × 10^− 54^	7.97 × 10^− 50^	0.85 (5.59 × 10^− 02^)	7.24 × 10^− 25^	1.18 × 10^− 20^
rs2314666	0.34	UBA52 (downstream)	0.85 (5.66 × 10^− 02^)	2.88 × 10^− 51^	3.81 × 10^− 47^	0.85 (5.73 × 10^− 02^)	3.41 × 10^− 24^	4.62 × 10^− 20^

*Adj. p value* is the adjusted *p* value with BH-correction; *MAF* denotes the minor allele frequency in this sample. The estimated Kendall’s *τ* between *SBP*_*adj*_ and *GE*_*UBA*52,*adj*_ is − 0.01. Note: The markers in the table are in very high linkage disequilibrium (LD) (with 0.96 ≤ *r* ≤ 0.99). SNVs rs6554 and rs10425018 in the same gene are in perfect LD with rs2314667 and have identical association; also rs7258480 is in perfect LD with rs2314664

After extracting the smallest *p* values among all SKAT and SKAT-O options in the *SKAT* function in R and applying a multiple testing correction for *p* values, 1 gene is identified to be associated with SBP (which contains the 5 significant variants identified under the copula model) and 36 genes are associated with GE. All of these genes are also identified in the single-variant analysis under the copula model. Table 3 shows the raw and adjusted *p* values of the top genes identified by SKAT and SKAT-O, and the minimum adjusted *p* values of all variants in or around the corresponding gene under the copula model for comparison. Much smaller *p* values are obtained using the single-variant analysis under the copula model incorporating multilevel biological measures.

**Table 3:** Raw and adjusted *p* values of the top genes identified by SKAT and SKAT-O and the minimum of the adjusted *p* values for variants in the gene under the copula model, obtained from testing *H*_0_ : *β*_1_ = 0 for GE

Gene	SKAT/SKAT-O		Copula	
	*p v*alue	Adjusted *p* value	*p v*alue	Adjusted *p* value
UBA52	1.36 × 10^− 12^	1.15 × 10^− 09^	6.47 × 10^− 60^	1.71 × 10^− 55^
IGFLR1	3.79 × 10^− 10^	1.61 × 10^− 07^	3.74 × 10^− 19^	1.24 × 10^− 15^
ACP5	3.12 × 10^− 09^	8.83 × 10^− 07^	4.95 × 10^− 12^	5.33 × 10^− 09^
CNN2	4.96 × 10^− 07^	9.06 × 10^− 05^	2.01 × 10^− 14^	3.56 × 10^− 11^
ANKRD27	5.34 × 10^− 07^	9.06 × 10^− 05^	6.08 × 10^− 13^	7.94 × 10^− 10^

**Fig. 3:**
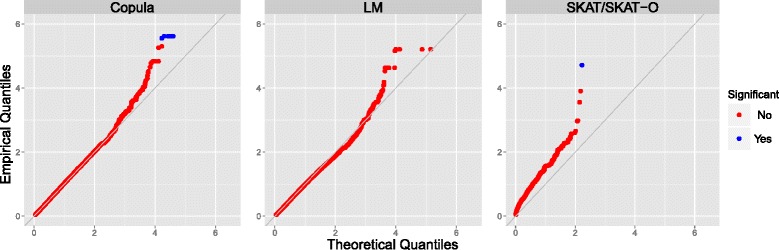
Uniform Q-Q plots of the *p* values (on a − log_10_ scale) for testing *H*_0_ : *α*_1_ = 0 under the copula model (*left panel*), univariate model (*middle panel*), and SKAT/SKAT-O (*right panel*). SNVs/genes which are significant after correcting for multiple testing are highlighted in *blue color*

The type I error of the test statistics, under the 3 modeling approaches, was checked using the observed data (see Fig. 3, for an illustration, showing quantile–quantile [Q-Q] plots of the *p* values for testing *H*_0_ : *α*_1_ = 0). The *p* values obtained under the copula model and the univariate regression models do not appear to show inflated type I errors, and the points corresponding to high *p* values lie on the diagonal line. There is some evidence for an inflated type I error for the gene-based SKAT and SKAT-O tests, when the minimum *p* value of all options in the *SKAT* package in R is extracted.

## Conclusions

A biological framework and genetic analysis approach based on a joint model of BP and GE is proposed, which contains a meaningful interpretation of possible biological relations. The approach, implemented in R, can be employed on a genome-wide scale and is illustrated in the analysis of 848 genes on chromosome 19. The analysis is computationally feasible and the association tests of all SNVs in the copula model took less than 1 day on four 32-core 2.6-GHz processors. Despite the small sample size and low dependence between SBP and GE, under the joint copula model, 5 rare variants in the gene *CEACAM5* are identified to be significantly associated with SBP, and in the eQTL analysis, 1075 variants in 122 genes are found to be significantly associated with their corresponding GE. More importantly, the Wald test statistic under the copula model seems to have a well-calibrated type I error and a higher power to detect both common and rare SNVs compared to univariate models and the well-recognized multimarker rare variant association tests, SKAT and SKAT-O. This underlines the potential usefulness of better phenotype modeling and of incorporating multi-level biological measures.

## Discussion

Because the focus was to implement and illustrate the analyses and interpretations within the proposed biological framework, as well as to show the potential usefulness of the approach by comparison to other approaches, the real data of unrelated individuals was used. The resulting small sample size can naturally limit the relevance of identified variants, as a consequence of the low power; however, it allows a more reliable comparison of the different methods without disentangling the effects when accounting for the family structure. For a more complete evaluation, a comparison of type I error rates has been conducted in an ongoing simulation study, and we observed that the Wald test statistics do not have inflated type I errors under the copula model. One restriction of the analyses was that rare variants with only 1 or 2 copies of the minor allele were not investigated under the copula model so that private mutations could not be detected. As a possible extension of the proposed approach, it would be interesting to include phenotypes on multiple levels (e.g., blood serum levels of biomarkers or epigenetic factors). Furthermore, measuring GE in cell types other than lymphocytes, for example in cells in the kidney, could lead to a higher association between SBP and GE. We anticipate that considering biologically meaningful complex joint models of multiple phenotypes will lead to more powerful association tests.
